# Effects of resistance training on body composition and functional capacity among sarcopenic obese residents in long-term care facilities: a preliminary study

**DOI:** 10.1186/s12877-018-0714-6

**Published:** 2018-01-22

**Authors:** Shu-Ching Chiu, Rong-Sen Yang, Rea-Jeng Yang, Shu-Fang Chang

**Affiliations:** 1 Department of Nursing, Central Taiwan University of Science and Technology, Taichung, Taiwan, Republic of China; 2 Department of Orthopaedics, National Taiwan University & Hospital, No. 7, Chung-Shan S. Rd, Taipei, Taiwan, Republic of China; 3 Department of Nursing, College of Nursing, National Taipei University of Nursing and Health Sciences, 365 Ming Te Road, Pei-Tou, Taipei, 112 Taiwan, Republic of China

**Keywords:** Sarcopenic obesity, Resistance training, Body composition, Activities of daily living, Grip strength, Long-term care

## Abstract

**Background:**

Aging-related loss of muscle and strength with increased adiposity is prevalent among older people in long-term care (LTC) facilities. Studies have shown that people with sarcopenic obesity (SO) are at high risk of declining physical performance. At present, no interventional studies on residents with SO in nursing homes have been conducted in the literature. The objectives of this study include appraising the changes in body composition and physical performance following resistance training among residents with SO in LTC facilities.

**Methods:**

This study used a quasiexperimental research design. Residents who are 60 years of age or above and have been living a sedentary lifestyle in LTC facilities for the past 3 months will be eligible for inclusion. The intervention group engaged in chair muscle strength training twice a week for 12 weeks, whereas the control group underwent the usual care. The main variables were physical parameters of being lean and fat, the strength of grip and pinch, and a functional independence measure using descriptive analysis, chi-squared test, t-test, and generalized estimating equation for statistical analysis through SPSS.

**Results:**

A total of 64 respondents with SO completed the study. After training, total grip strength (*p* = 0.001) and total pinch strength (*p* = 0.014) of the intervention group differed significantly from those of the control group. The right grip strength of the intervention group increased by 1.71 kg (*p* = 0.003) and the left grip strength improved by 1.35 kg (*p* = 0.028) compared with baseline values. The self-care scores of the intervention group increased by 2.76 points over baseline scores, particularly for the action of dressing oneself. Although grip strength and self-care scores improved more among those in the intervention group, body fat and skeletal muscle percentages did not differ significantly between the groups after training (*p* > 0.05).

**Conclusions:**

Resistance exercises for elderly residents in LTC facilities may play an important role in helping them maintain physical well-being and improve muscle strength.

**Trial registration:**

Clinicaltrials.gov, number NCT02912338. Retrospectively registered on 09/21/2016.

## Background

Sarcopenic obesity (SO), one of the physiological changes and associated health risks of aging, is characterized by a combination of low muscle mass and strength combined with high fat mass and is negatively correlated with functional independence [1, 2]. Residents of long-term care (LTC) facilities have a high prevalence of SO due to advanced age as well as long-term sedentary behavior [3–5]. Mitochondrial dysfunctions are signaling pathways in the pathogenesis of skeletal muscle wasting with disuse atrophy, whereas enhancing mitochondrial antioxidant activity results in the enhancement of skeletal muscle function and voluntary motion in elderly individuals [6, 7]. Exercise causes muscles to release myokines, which can protect against age-associated diseases with antioxidant activity [8, 9].

Resistance training is an important intervention for elderly people in nursing homes [10]. It can enhance lean tissue, muscle strength, and functional performance [11]. However, effects of resistance training on SO have not been clearly quantified, as only a few resistance training studies have been specifically designed for SO [12–16]. In community studies, two studies have reported that resistance training can increase grip strength [12, 15], one study reported an increase of lean mass [13], one study reported a reduction of body fat mass [14], and one study declared that the effects were statistically insignificant [16]. However, none of these studies were conducted for elderly people with SO in nursing homes.

Prevalence of SO among elderly people in the general population has been estimated at 15%–30% [17, 18]. For residents of nursing homes, the rate of SO is estimated at 22% [5]. Particularly, 37% had low muscle mass, 86% had low gait speed, 95% had low grip strength [19], and more than 40% were overweight or obese [5] in nursing homes. Furthermore, more than 80% of the nursing home residents had functional limitations and comorbidities [20]. A review study highlighted the common prevalence of elderly with inadequate exercise in LTC facilities and the lack of interventional studies [21]. In the present study, elderly people with SO in LTC facilities were assumed to live a sedentary lifestyle and have lower functional capacity, thereby necessitating program intervention. Thus, the purpose of the study was to determine the effects of resistance training on body composition, strength of grip and pinch, and Functional Independence Measure (FIM) scores among LTC residents with SO with sedentary lifestyles.

## Methods

### Study design

A 12-week quasiexperimental study with intervention and comparison groups was conducted to determine the effects of chair resistance training on body composition and physical performance among SO residents of nursing homes. Considering the practical limitations, a nonrandomized study design was applied because we could not quantify the number of individuals in order to randomly assign facilities or clients to groups due to the weak epidemiological evidence for investigating elderly with SO in the nursing homes [5]. Therefore, to ensure high design quality, the statement of transparent reporting of evaluations with nonrandomized designs was used as a guide in this study to improve the reporting standards of nonrandomized evaluations of health interventions [22].

### Participants and setting

The eligibility criteria for inclusion were residents 60 years or older, those who had no limited exercise recommendation from a physician, and those living continuously in LTC facilities for the past 3 months. The respondents lived a sedentary lifestyle (defined as exercising less than 150 min in a week) and could sit independently for at least an hour on the edge of a bed as well as understand Chinese or Taiwanese and follow instructions. Exclusion criteria included fluctuating weight (> ± 3 kg) for the past 3 months, limbs completely unable to resist gravity, severe cognitive impairments (clinical dementia rating ≧ 3), and severe disease status such as severe cardiopulmonary liver and kidney diseases, severe malignancy, and undernutrition (Mini Nutritional Assessment scale of 0–7 points). The study received support from the senior administrator of the LTC facilities involved. During preparation, the senior health leader of the LTC facilities helped screen or introduce elderly residents for this study. All subjects also gave oral and written informed consent prior to inclusion in the study.

The settings included six LTC facilities in Taichung City, Taiwan, a city (2215 km^2^) with an elderly population of nearly 310,000. We selected three facilities in urban areas and three in suburban areas. Participants were recruited from three Ren-ai Senior Citizens’ Homes and three nursing homes. The resistance training was implemented between October 2015 and March 2016. Blinding of the two groups for participants was not established, but the subjects and professional health trainer had been blinded to either SO or non-SO residents. Before assessment, the individuals making the relevant measurements had been instructed with the study’s training guide, ensuring better interrater and intrarater reliability. Each training session had 5–15 elderly participants and was held in a lounge or an activity room depending on the setting situation of the institution.

### SO identification

The identification of SO was from a combination of sarcopenia and obesity. For sarcopenia, the method was based on the equation: skeletal muscle mass ÷ body mass × 100 [23]. For men and women, the cutoff values for sarcopenia (two standard deviations (SDs) below the mean for the young reference group) were 37.15 and 32.26%, respectively, as previously reported in a Shanghai population study [24]. For obesity, the body mass index corresponding to the 90th percentile of body fat was 25.4–26.1 kg/m^2^; the obesity cutoff points by percent of body fat in men and women were 29 and 40%, respectively, following a Hong Kong population study [25].

### Intervention

For the intervention group, the resistance training intervention program emphasized chair muscle strength training using 2–5 lbs. sandbags on wrist or ankle joints and a grip ball. The comparison group comprised participants who signed the consent letter for join the study but as unwilling to participate in resistance training. Design ideas were drawn from resistance exercises for physical fitness enhancement of sarcopenia and program reference supported from Chang [26]. To motivate the participants, a professional health trainer added nostalgic music at a tempo matched with resistance training and played games with them. The training program had consulted with a multidisciplinary healthcare team that included nurses, a physiotherapist, an occupational therapist, and sports experts. During the first day of training, the respondents did the exercises without load to adjust themselves to the movements and posture.

All intervention groups trained with two sessions each week for a total of 3 months. An interval of at least 48 h between exercise sessions was arranged. The entire, the approximately 60-min exercise consists of the following three parts: warm-up stage, muscle resistance training while sitting, and relaxation stage. The intervention program comprised both upper body and lower extremities training. Upper body exercises included training that targeted the biceps, deltoids, grip, and pinch. Lower extremities consisted of leg extension, leg flexion, calf raises, stepping forward and sideward, and others. All exercises were performed for three sets of 4–10 repetitions with about 30 s rest between each set (Table 1).

**Table 1:** Schedule of seated resistance training per section

Item	Interventional protocol	Tracks/ Temple	Body movement patterns	Rep	Set
Warm-Up 5 mins	Basic actives without loads	Piano /Adagio	Range of motion for different joints from head to toe. Arm curl, hands up, knee extension and knee flexion, stepping.		
Strength training 5 mins	Basic movements with loads	Piano /Adagio	Arm curl	6	1
			Hand up	4	
			Arm lateral raise	4	
			knee extension	4	
			calfraises	4	
			stepping	4	
Strength training 7–10 min	Upper extremities with loads	Kids song /Andante3:30	biceps curl	8	3
			hand up	6	
			Arm lateral raise and Inversion	2	
			Arm lateral raise and Eversion	2	
			Stepping	8	
			boxing	8	
Strength training 4–6 min	Upper extremities with loads	Reminiscing song /Andante2:05	Arm front raise	4	3
			Arm Adduction and abduction	6	
			Hand up	10	
			biceps curl	4	
Strength training 5–7 min	Lower extremities with load	Reminiscing song /Andante2:35	Hip flexion	8	3
			knee extension	8	
			foot dorsiflexion	8	
			hand push	8	
			knee extension	8	
Game 10–15 min	Play/Competition with or without loads	Background music	Clap balls, toss balls, pass balls, catch a ball, kick a ball, grip a ball and pinch a ball with fingers	6	3
Cool down 5 mins		Background music	Relaxing all muscles from head to toe, a gentle stepping, a light static stretching, some slowly and deep breath		

Rep, Repetition; all exercises were performed with approximately 30 s of rest between sets. Ankle and wrist weights had loads ranging from 2 to 5 lbs

Before giving them weights, we assessed the residents’ condition through their vital signs, investigated their health history (such as skin wounds, disease, and any operations), and checked their grip strength. During the training process, we encouraged participants to provide feedback and other information. After the session, we recorded the load level of the rating scale of perceived exertion (RPE) and respected residents’ decisions regarding the suitability of each sandbag weight. RPE is an objective method for quantifying the intensity of resistance exercise. RPE is a psychophysical scaling with a score between 6 and 20 points, with 9 points standing for very light, 11 for fairly light, 13 for somewhat hard, 15 for hard, and 17 for very hard [27].

### Measurements

The Cumulative Illness Rating Scale (CIRS) is an objective and simple method of assessing physical impairment. This index measures chronic medical illness burden while taking into consideration the severity of chronic diseases. The scale format provides for 13 relatively independent areas grouped under body systems. Ratings are made on a 5-point degree of severity scale, ranging from “none” to “extremely severe” [28]. On this scale, 0 = no problem affecting that system, 2 = moderate disability or morbidity or requires first line therapy, 4 = extremely severe problem or immediate treatment required or organ failure or severe functional impairment [29].

As a portable, noninvasive method appropriate for persons with disabilities, bioimpedance analysis (BIA) estimates body composition of fat and lean tissue [30]. The measurements were made according to InBody’s instruction manual for BIA (InBody S10, Biospace, Seoul, Republic of Korea). BIA was performed with eight surface electrodes placed on a resident’s thumbs, middle fingers, and both sides of each ankle with six different frequencies of 1, 5, 50, 250, 500, and 1000 kHz [31]. The InBody uses segmental analysis can determine differences caused by gender, aging, disease, and ethnicity without any empirical estimation. Exclusion criteria were the contraindications of the device: presence of an electronic implant, such as a pacemaker, artificial heart/lung, or portable electrocardiograph [31]. One resident with a heart pacemaker was excluded from the study.

Isometric hand grip strength was measured using a Jamar Lafayette hydraulic hand dynamometer (J00105) with residents in a sitting position holding the dynamometer at approximately 90° flexion to their elbow. The hand dynamometer used is reliable and widely cited in the literature [32]. The finger pinch gauge can be used to measure pinch strength [33] by applying pinch force at the pinch groove while holding the pinch gauge between one’s thumb and finger(s) using a Lafayette Hydraulic pinch gauge (5030P1). For grip and pinch strength, maximal readings of three measurements from both the left and right hands were recorded [34, 35]. Total handgrip strength was summed up from readings of both hands. Grip and pinch strength were presented in kilograms of force units.

The FIM scale was designed to measure one’s ability to function with independence [36]. FIM included 18 items, covering six situations in the burden of personal care, specifically self-care, mobility, transfers, sphincter management, communication, and social cognition. A high score indicates better function. If the subjects’ functional status varies in different settings or at different times of the day, the lower rating was recorded in accordance to the principles outlined in the FIM system clinical guide [36]. A high test–retest reliability for residents repeating the FIM assessment for the motor intraclass correlation (ICC = 0.9) and cognitive subscales (ICC = 0.8) was demonstrated along with construct validity of FIM [37].

### Power and sample size

The sample sizes for the study was calculated based on the muscle strength variable using G-Power 3.1 software with 80% power and an alpha of 0.05. Effect size was found with the standardized mean difference of 0.68 (95% CI 0.52 to 0.84) from systematic literature [38]. Each group included 35 participants with a total of 70 subjects at baseline. After 3 months, 91.4% subjects (*n* = 64) had completed the study. Our research team recalculated the effect size to be 0.76 and a power of 0.85 based on the grip strength variable.

### Statistical analysis

All the collected data were subjected to descriptive and frequency analyses, using the baseline of the two groups’ differences through chi-squared test and t-test. Additionally, generalized estimating equation (GEE) was used to analyze the repeated measurements of independent effectiveness on the independent variables [39]. All data were processed in SPSS version 17.0 (SPSS Inc., Chicago, IL, U.S.A.). A *p* < 0.05 was considered statistically significant. For missing data, we used simple imputation to deal with this issue, including last or baseline observation carried forward as close to real-time as possible during the study [40].

## Results

**Fig. 1:**
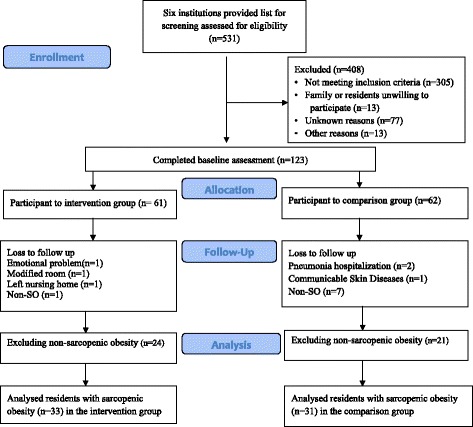
Flowchart of the quasiexperiment showing nonrandomization and number of participants

At the end of the 3-month intervention, 33 participants in the intervention group and 31 in the comparison group completed all the measures from the six LTC institutions. More women joined the interventional group compared with men (*p* = 0.056). Overall, 8.6% (6 of 70) loss was observed in the follow-up for participants (Fig. 1). In our study, the intervention cohort had 66.7% (*n* = 22) completion for over half of the training days and 24.2% (*n* = 8) completion for at least three quarters of the program content. Participation rate in this study was 80.8% (19.4/24). More male residents than female residents had missing data; male residents also had slightly higher on grip strength, higher functional status, and lower body fat percentage, but these differences were not statistically significant.

The participants’ mean age was 79.9 years (SD = 7.8, range of 63–96) and 50% of the sample were females. In addition, 42.9% of the participants had never been educated and 28.1% of the respondents were nonreligious people. The mean of the summary CIRS scores was 2.73 (SD = 1.20) and the prevalence of hypertension, diabetes, and heart disease were 48.6, 34.3 and 28.6%, respectively. Apart from the total FIM score, no further significant group differences in the preassessment were observed (Table 2).

**Table 2:** Demographic and baseline characteristics of subjects between two groups

	Intervention Group	Comparison Group	*χ2/t*	*p*
Gender (M/F)	14/22	21/13	3.660	0.056
Age(yrs.) (SD)	79.64(7.36)	80.15 (8.26)	0.272	0.786
Education (no/yes)	14/22	16/18	0.477	0.490
Religion (no/yes)	8/28	13/21	2.135	0.144
CIRS total (SD)	2.76 (1.20)	2.71 (1.22)	−0.159	0.875
	Mean (SD)	Mean (SD)	t	p
Body composition				
Body weight (kg)	59.63 (10.52)	61.19 (8.17)	0.693	0.491
BMI(kg/m^2^)	25.15 (3.75)	24.85 (3.01)	−0.364	0.717
Fat mass (kg)	25.23 (6.76)	24.56 (4.72)	−0.474	0.637
Fat (%)	42.07 (6.00)	40.29 (6.87)	−1.149	0.255
Fat free mass(kg)	34.26 (5.99)	36.64 (6.96)	1.235	0.132
Soft lean mass(kg)	32.33 (5.78)	34.57 (6.67)	1.494	0.140
Skeletal muscle mass(kg)	17.79 (3.54)	19.09 (4.06)	1.413	0.162
Skeletal muscle (%)	29.98 (3.44)	31.03 (4.11)	1.155	0.252
Skeletal muscle index (kg/m^2^)	7.48 (1.02)	7.68 (1.18)	0.732	0.467
Lean mass index (kg/m^2^)	13.61 (1.58)	13.92 (1.87)	0.751	0.455
ASM (kg)	13.66 (3.43)	14.92 (3.85)	1.437	0.155
ASM (%)	22.81 (3.31)	24.15 (4.28)	1.465	0.148
ASMI(kg/m^2^)	5.72 (1.07)	5.98 (1.19)	0.964	0.338
Physical performance				
^a^Total grip strength(kg)	27.29 (13.76)	24.67 (17.34)	0.699	0.487
^a^Total pinch strength(kg)	5.28 (3.02)	4.86 (3.85)	0.507	0.614
Total FIM score	112.67 (17.89)	82.09 (33.79)	4.692	< 0.001^***^
FIM self-care	35.89(9.12)	25.41(12.51)	3.986	< 0.001^***^

*ASM* appendicular skeletal muscle mass, *ASMI* appendicular skeletal muscle mass index, *BMI* body mass index, *CIRS* Cumulative Illness Rating Scale, *FIM* functional independence measure

^a^Total grip/pinch strength was summed up from readings of both hands

^***^P < 0.001

**Fig. 2:**
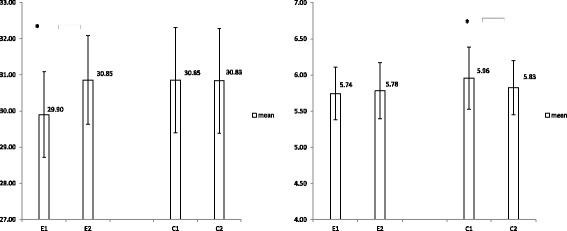
Mean and 95% CI of percentage of skeletal mass (a, left) and appendicular skeletal muscle mass index (b, right); changes between groups and time interaction using GEE statistical analysis. Asterisks indicate significant within-group differences, ^*^p < 0.05

After 3 months, SO phenotypes from the intervention group had decreased by 8.9% compared with the control group (12.1% versus 3.2%, *p* > 0.05). No significant between-group differences were observed for any of the body composition outcomes. However, three significant within-group differences were observed for skeletal muscle, appendicular skeletal muscle mass (ASM), and ASM index (ASMI) at 3 months. Participants in the intervention group improved their skeletal muscle by 0.95% on average (*p* = 0.035) in relation to baseline values. Participants in the comparison group decreased their mean ASM and ASMI by 0.34 kg and 0.13 (kg/m^2^) (*p* < 0.05), respectively, on average in relation to baseline values (Table 3, Fig. 2).

**Table 3:** Effect of chair resistant training intervention on body composition and physical performances using GEE

	Baseline	3 months	Within-group differences		Between-group differences	
	*Mean(SE)*	*Mean(SE)*	*Mean (SE)*	*p*	B (95%CI)	*p*
Body composition						
Skeletal muscle (%)					0.973(−0.018 to 1.963)	0.054
Intervention group	29.90(0.60)	30.85(0.62)	0.95(0.45)	0.035^*^		
Comparison group	30.85(0.74)	30.83(0.74)	−0.02(0.23)	0.931		
ASM (kg)					0.453(−0.039 to 0.946)	0.071
Intervention group	13.75(0.60)	13.87 (0.64)	0.12(0.20)	0.550		
Comparison group	14.82(0.71)	14.48(0.64)	−0.34(0.16)	0.034^*^		
ASMI (kg/m^2^)					0.169(−0.019 to 0.358)	0.078
Intervention group	5.74(0.19)	5.78(0.20)	0.04(0.07)	0.603		
Comparison group	5.96(0.22)	5.83(0.19)	−0.13(0.06)	0.031^*^		
Body fat (%)					−1.498(−3.259to0.262)	0.095
Intervention group	42.29 (1.05)	40.76 (1.07)	−1.53(0.79)	0.053		
Comparison group	40.64 (1.23)	40.61 (1.24)	−0.03(0.43)	0.940		
Physical performances						
Total grip strength^a^ (kg)					7.163(2.859 to 11.47)	0.001^**^
Intervention group	26.77(2.42)	29.77(2.28)	3.0(0.89)	0.001^**^		
Comparison group	24.56(3.20)	20.40(3.08)	−4.16(2.01)	0.038^*^		
Total pinch strength^a^ (kg)					1.462(0.302 to 2.622)	0.014^*^
Intervention group	5.19(0.53)	5.31(0.50)	0.12(0.34)	0.720		
Comparison group	4.88(0.71)	3.54(0.55)	−1.34(0.49)	0.006^**^		
Total FIM score^b^					0.178(− 1.461 to 1.817)	0.832
Intervention group	96.65(0.12)	96.90(0.17)	0.24(0.11)	0.033^*^		
Comparison group	96.30(0.12)	96.37(0.83)	0.06(0.83)	0.938		
FIM self-care^b^					1.951(−0.83, 4.73)	0.169
Intervention group	30.51(0.19)	33.26(1.19)	2.76(1.08)	0.011^*^		
Comparison group	29.91(0.24)	30.72(0.92)	0.81(0.91)	0.378		

*ASM* appendicular skeletal muscle mass, *ASMI* appendicular skeletal muscle mass index, *FIM* functional independence measure

^a^Total grip/pinch strength was summed up from readings of both hands

^b^Adjusted mean (SE) changes from baseline

*P* < 0.05,^**^P < 0.01

**Fig. 3:**
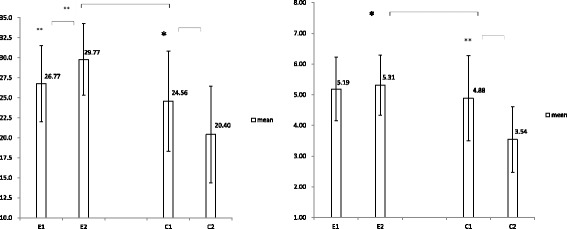
Mean and 95% CI of total grip strength (a, left) and total pinch strength (b, right); changes between groups and time interaction using GEE statistical analysis. Asterisks indicate significant between-group and within-group differences (^*^p < 0.05, ^**^ *p* < 0.01)

**Fig. 4:**
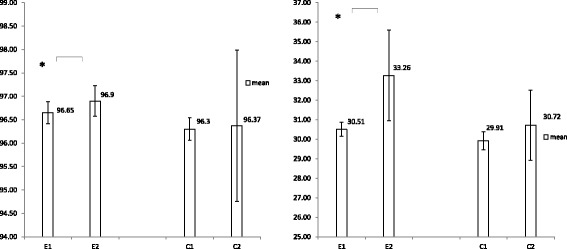
Mean and 95% CI of the functional independence measure (a, left) and self- care (b, right); changes between groups and time interaction using adjusted GEE statistical analysis. Asterisks indicate significant within-group differences, ^*^p < 0.05

Between-group analysis revealed significant differences over time for total grip and pinch strength (*p* = 0.001, *p* = 0.014), with the intervention group increasing strength and the comparison group decreasing (Table 3, Fig. 3). However, no significant between-group differences were observed for the total FIM score and self-care, despite the significance in within-group differences (*p* = 0.033; Table 3, Fig. 4) using adjusted GEE analysis.

## Discussion

This study is the first to utilize an intervention design employed for LTC residents in Asia with SO. The cutoff values selected for skeletal muscle mass and body fat percentages were based on previous studies concerning race and health hazards, along with issues on determining the best measures to prevent high prevalence of comorbidities and reduce mortality [23–25, 41]. One study supported of the use of skeletal muscle mass percentage for screening sarcopenia in more at-risk populations rather than skeletal muscle mass normalized for height [42].

In our study, women with SO had higher body fat percentages (~ 11%) and lower skeletal muscle percentages (~ 6%)/skeletal muscle index (~ 1) compared with men, which is consistent with the current literature [43]. Moreover, the elderly with SO had higher waist circumferences (~ 7.5 cm) and lower appendicular skeletal muscle masses (~ 3.6 kg)/skeletal muscle index (~ 0.9) compared with the Shanghai elderly [44].

Using resistance training for the purpose of changing body composition is not an easy task among elderly. The two study groups were statistically nonsignificant in their difference in skeletal muscle mass despite the fact that patients could maintain lean body mass after exercise training [12, 15]. Training duration may be the main reason for this result. One study stated that resistance training for 24 weeks is an effective approach to improve lean tissue [13].

However, a minor effect on body fat was observed in this study, which was similar to other interventional studies [12, 13]. The mechanisms of pathophysiological transformation regarding adipogenic cells and muscular growth have remained unclear [45]. One study suggested that exercise combined with adjusted food composition using increased dietary protein, as well as green tea, to reduce fat intake may be a good approach for elderly living in the community [14].

Our study offers evidence that resistance training with a sandbag can increase muscle strength among elderly people, including those with disabilities. The mean maximum grip strength improved by 1.53 kg after training, which is an outcome consistent with three other papers [12, 15, 46]. From the training process, we found that total grip strength among those in the intervention group was influenced by hand load. Hand load is equal to the number of participants × sandbag weight (lb) × time (minutes). Most of the participants showed the rate of 12–13 points of RPE with sandbag loads of about 1.3%–3.5% of body weight in our study. Relevant literature shows that lower weight loads could be beneficial for elderly in promoting exercise over a longer period of time [12].

The main training effect of total grip strength was influenced by a number of factors, which included demographic characteristics of residents with SO using GEE analysis interventional data, statistically significant differences followed by order CIRS, gender, age, and education (Table 4). Moderate disability or morbidity was the most important demographic factor for total grip strength among residents of LTC facilities. Females has less total grip strength (~ 10 kg) than did males and residents over 75 years of age had less total grip strength (~ 7 kg) than did those in the 60–75-year-old range. One previous study stated that grip strength performance was associated with numerous variables, such as gender, aging, stress, physical activity at work, and chronic diseases [47]. However, total grip strength was not influenced by the factor of religion.

**Table 4:** Generalized estimating equation for the main effects on total grip strength associated with demographic characteristics among the intervention subjects

	Wald				95% CI	
Parameter	Estimate	SE	Chi-Square	p	upper	lower
Intercept	39.59	4.64	72.813	< 0.001^***^	30.495	48.681
Time(T2 vs T1)	3.00	0.89	11.424	0.001^**^	1.261	4.742
Age(above 75 vs 60-75 years old)	−7.01	3.53	3.945	0.047^*^	−13.929	−0.092
Gender(Female vs Male)	−9.61	3.83	6.312	0.012^*^	−17.111	−2.113
Education(Yes vs No)	6.70	3.28	4.163	0.041^*^	0.264	13.139
CIRS (≧2.5 vs < 2.5)	−10.39	3.35	9.638	0.002^**^	−16.951	−3.831

*SE* standard error, *95% CI* 95% wald confidence interval, *CIRS* cumulative illness rating scale

*p < 0.05 **p < 0.01 ***p < 0.001

For the self-care subscale of the FIM, the intervention group increased by 2.76 points and the control group increased by only 0.81 points. Among them, the scores of lower body dressing improved the most, followed by upper body dressing and bathing. Other subscales of FIM, such as transfers, sphincter control, locomotion, communication, and social cognition did not have statistically significant differences. A systematic review paper described that progressive resistance training is a valid program for preserving independence levels by reducing physical disability in performing the activities of daily living in LTC institutions [10]. However, other papers showed that a strength training program does not improve functional capacity or that no consistent effect is observed on the daily living function [12, 48, 49]. Inconsistent results may be due to whether the training design was connected to specific daily living functions and other factors that may have had an effect, such a disease status.

For group activities, our researcher team are not surprised that more women desired to participate in the interventional group compared with men who are unfamiliar with group exercises. Women favored different types of activities and social pursuits compared with men [50]. However, men had more positive attitudes toward the loads of sandbag compared with women. Considering gender issues, we incorporate music and games into our study for fun, which led to a positive result. The elderly and their families favored the training process.

Some study limitations were observed. First, we excluded some bedridden residents with disability or severe disease status, which could affect external validity. Second, this study was conducted in six LTC institutions using a quasiexperimental design and implemented the intervention program in a natural environment. The functional status of the comparison participants was generally poor to pay reverence to the will or living habits of the elderly, and two participants were lost to follow-up because of their medical condition (pneumonia) that required hospitalization. While the attrition rate of this study was less than 10%, it could have a major impact on a study with a small sample size.

Third, our method of measuring body composition may not be the best. In the literature, compared with BIA, dual energy X-ray absorptiometry (DEXA) and computed tomography appear to be more reliable tools for measuring body composition [30, 51]. However, these large and fixed tools are unsuitable for residents in LTC facilities. Although the reliability of the test–retest of the BIA was ICC 0.7 for inter-rater reliability and ICC 0.89 for intra-rater reliability [30], the high correlation (r ~ 0.9) of body composition between BIA and DEXA [43] could provide a simple, portable, reliable, and low-cost alternative.

Only a few previous studies have discussed the effects of metal prostheses on body composition measurement. One study stated the lack of agreement between BIA and DEXA was not due to the presence of metal prostheses or certain diagnoses, such as hypertension and edema [52]. Another paper studying the effects of metal on DEXA found that the presence of metal rods weighing 0.1 kg significantly increased reported total body mass and bone mineral content and soft-tissue mass [53]. Another paper showed that, in repeated DEXA scans of a same individual, the errors in body composition induced by the metal are reproducible and will not reduce its ability to detect change [53].

## Conclusions

This study found that twice-a-week chair muscle training with sandbags was feasible for LTC residents with SO. Our findings indicate that this training regimen can help individuals maintain skeletal muscle mass and significantly improve total grip and pinch strength. Resistance training further promotes self-care ability. Furthermore, our findings suggest that regularly participating in resistance training with engaging small group activities is beneficial for the residents of LTC facilities.

## Abbreviations

95% CI: 95% Confidence interval
ASMI: Skeletal muscle mass index
BIA: Bioimpedance analysis
CIRS: Cumulative illness rating scale
DEXA: dual energy X-ray absorptiometry
FIM: Functional independence measure
GEE: Generalized estimating equation
LTC: Long-term care
RPE: Rating of perceived exertion
SO: Sarcopeic Obesity
